# Production of Biocalcium from Fermented Fish Bone Residue for Fish Emulsion Sausage Fortification

**DOI:** 10.3390/foods13060882

**Published:** 2024-03-14

**Authors:** Somsamorn Gawborisut, Chavis Ketkaew, Thongsa Buasook

**Affiliations:** 1 Fish Processing Laboratory, Faculty of Agriculture, Khon Kaen University, 123 Mittraphap Rd., Khon Kaen 40002, Thailand; 2 International College, Khon Kaen University, 123 Mittraphap Rd., Khon Kaen 40002, Thailand; chaket@kku.ac.th; 3 Research Center and Central Laboratory, Faculty of Agriculture, Khon Kaen University, 123 Mittraphap Rd., Khon Kaen 40002, Thailand; thonbu@kku.ac.th

**Keywords:** biocalcium, fermented fish bone residue, alkaline treatment, calcium, fish emulsion sausage, by-product

## Abstract

Fermented fish bone residue (FFBR) is an underused by-product of the industrial-scale production of fermented fish sauce. Subjecting FFBR to proper alkaline treatment can transform FFBR into biocalcium, which can be added to fish emulsion sausage (FES) to increase its calcium content. This study comprised two experiments. First, we aimed to find the most suitable alkaline treatment conditions for preparing biocalcium from FFBR. Alkaline treatments combining three sodium hydroxide (NaOH) concentrations (0%, 3%, and 6%) and three soaking times (0, 1, and 2 h) were tested. Quality parameters of alkaline-treated biocalcium (crude protein, crude fat, ash content, calcium, phosphorus, crude fiber, salt content, CIE color values, morphology of biocalcium particles, and the intensity of the fermented fish smell) were assessed. Second, we fortified FES with the properly treated biocalcium (0, 12, 24, or 36 g) and evaluated the sausage’s calcium, phosphorus, crude fiber, salt content, pH, CIE color values, texture profile analysis (TPA), emulsion stability, and sensory criteria. It was found that treatment with 3% or 6% NaOH produced better crude protein, ash content, calcium, and CIE color value results than no alkaline treatment. These two NaOH concentrations effectively lowered the salt content and the intensity of the fermented fish smell. However, 3% and 6% NaOH produced similar results. A soaking time of 1 h or 2 h produced better results than no soaking in terms of crude protein, crude fat, ash content, calcium, phosphorus, CIE color values, and the intensity of fermented fish smell. However, 1 h and 2 h produced similar results. It is concluded that 3% NaOH and soaking for 1 h would be the most suitable alkaline treatment to prepare biocalcium from FFBR. Fortifying FES with biocalcium from FFBR increased the calcium and phosphorus contents but slightly reduced TPA. The other FES quality parameters were unaffected by biocalcium fortification.

## 1. Introduction

Fermented fish is one of the most important food staples in Southeast Asian countries. It is a mixture of fish, salt, and roasted rice/rice bran tightly packed in earthenware jars and stored for at least 6 months in the shade [1]. In the past, people in Southeast Asia had used fermented fish as a main dish and condiment. However, at present, they use fermented fish sauce stored in lightweight plastic bottles; it is convenient to use and can be transported well. Fermented fish sauce is manufactured by simmering ripened fermented fish in a large stainless-steel pan for 24 h. Salt and water are generally added to adjust the volume and salinity of the simmered fermented fish [2]. The well-hydrolyzed fish meat in fermented fish is solubilized during simmering and transformed into meat particles suspended in the liquid. Fish bones originating from fish bodies in fermented fish settle at the bottom of the pans, and they need to be removed regularly. The removed fish bones become a by-product called fermented fish bone residue (FFBR). After simmering, the liquid in the pans is pumped through filters to separate small fish bones and rice residues. The filtered liquid is used to manufacture fermented fish sauce. It is generally combined with sugar and flavor-enhancing ingredients, pasteurized, and bottled [3]. The filtration process yields another by-product called rice residue. In general, the FFBR-to-rice residue ratio is approximately 60:40 by weight. These residues are generally combined and called fermented fish residues.

It is estimated that the fermented fish sauce industry discards fermented fish residue of about 676–1352 tonnes/year [2]. Therefore, around 406–811 tonnes of FFBR are discarded each year. This by-product will continue to accumulate when fermented fish sauce production expands. FFBR is an underutilized by-product because of its high salt content of 9.16 g/100 g and pungent fermented fish smell [2]. It is mostly dumped in large concrete ponds in remote areas to prevent salt from sieving into the surrounding soil and to limit exposure to its undesirable smell. Gawborisut and Huaisan [2] have reported that fresh FFBR contains a high calcium content (7.57 g/100 g) and that, after being dried, the calcium content increased to 15.38 g/100 g. Due to this high content, FFBR has the potential to be used as biocalcium.

Before FFBR can be used as biocalcium, its high salt content, pungent fermented fish smell, and dark color need to be addressed. Water treatment can be used to reduce the salt content in FFBR. Our preliminary study showed that washing FFBR twice in a stirrer using a FFBR-to-tap water ratio of 1:2 (*w*/*v*) for 15 min could reduce the salt content in the residual from 9.16 to 2.01 g/100 g [2]. The washing could also increase levels of calcium and phosphorus in FFBR to 10.46 and 5.83 g/100 g, respectively [2]. After that, the residue must be sterilized in an autoclave at 120 °C for 1 h to brittle the bones and to vaporize some of the volatile compounds that produce the fermented fish smell. Then, FFBR is dried at 100 °C in a hot air oven, pulverized into powder, sieved, and kept in plastic bags in ambient conditions.

Although FFBR was subjected to a series of treatments, it still had an undesirable brown color and a fermented fish smell [2]. It also contained 9.38% protein and 0.20% fat [2]. These preliminary results suggest that additional alkaline treatment is required. Theoretically, alkaline treatment is believed to reduce protein and fat in FFBR powder. Alkaline treatment can effectively solubilize protein and saponify fat, thus rendering them from fish bones [4,5]. The treatment relies on submerging fish bones in alkaline media, mostly sodium hydroxide (NaOH). During the treatment, the temperature of the alkaline solution may be elevated to increase protein solubility and fat saponification. Researchers have used alkaline media to remove protein in tuna bones from the tuna canning industry and fish bones from the fish filleting industry [5,6,7,8]. Various concentrations of NaOH or potassium hydroxide (KOH) (0.8–2%) and soaking times (0.5–2 h) at elevated temperatures (80–100 °C) have been used in combination to treat the bones. These treatments could remove crude protein and fat in fish bones and could increase calcium and phosphorus in the bones to >20% and 10%, respectively. Thus, alkaline treatment can effectively remove proteinaceous impurities in the bone and may be used commercially due to its viability in local and international markets and its affordable price (2–3 USD/kg food grade).

Although alkaline media has been used in several studies to treat fish bone, none of the experiments have been conducted on FFBR containing both protein and an unpleasant color and smell. Our preliminary studies show that using low NaOH concentrations (1% and 2%) and short soaking times (15, 30, and 45 min) partially removed protein, fat, and dark color but ineffectively removed fermented fish smell. Higher alkaline concentrations alongside longer soaking times may be required to remove these undesirable components.

After alkaline treatment, FFBR is transformed into biocalcium, which can be used to increase the calcium content of foods. Biocalcium fortification in foods such as cookies, cakes, rice crackers, noodles, pork emulsion sausage, candy, and biscuits has been investigated [4,9,10,11,12,13,14,15,16,17]. There have been several studies on fortifying fish-based gel products such as fish emulsion sausage (FES), surimi, fish tofu, and fish balls with biocalcium powder or fish bone extract [18,19,20,21,22,23,24,25,26].

Biocalcium fortification in these products could increase calcium levels by 5–10 fold depending on the amount of biocalcium added to the products [18,22,24]. In addition, the fortification tended to improve the instrument texture of the products [18,19,21,22,23,24,25]. Color improvement in fish-based gel products after biocalcium fortification depended on the color of the biocalcium and that of the product. Fortification of light or off-white biocalcium in light color products such as surimi and FES could improve their lightness quality [19,22,23].

To our knowledge, there has been no study on FES fortification with biocalcium produced from FFBR. Hence, we addressed this research gap. We divided this study into two experiments. First, we determined the NaOH concentration and soaking time that yielded the highest quality biocalcium from FFBR. Second, we fortified FES with properly treated biocalcium and investigated the effect of this fortification on the quality parameters of the product.

## 2. Materials and Methods

### 2.1. Investigation of the NaOH Concentration and Soaking Time That Yield the Highest Quality Biocalcium from FFBR

#### 2.1.1. Preliminary Treatment of FFBR

FFBR was obtained from a fermented fish sauce manufacturer in Kalasin province, Thailand. Upon arrival, FFBR was placed into a stirrer with a maximum capacity of 100 kg. FFBR was mixed with tap water at a ratio of 1:2 (*w*/*v*). The mixture was then stirred continuously at 70 rpm for 15 min. Subsequently, FFBS was strained using a plastic net and rewashed for another 15 min using the same FFBR-to-tap water ratio. The washed residue was strained, sterilized in an autoclave at 120 °C for 1 h, dried at 100 °C for 3 h, observed under a scanning electron microscope, pulverized into powder using a 500 g multi-function disintegrator WF-10B (Zhejiang Horus Industry and Trade Co., Ltd. Yongkang, Zhejiang, China), sieved with a stainless-steel strainer with a 425-μm sieve, and kept in plastic bags at ambient conditions. This prepared FFBR powder was subsequently subjected to alkaline treatment.

#### 2.1.2. Alkaline Treatment and Biocalcium Preparation

Three NaOH concentrations (0%, 3%, and 6%) and three soaking times (0, 1, and 2 h) were used in combination to treat FFBR powder. The prepared FFBR powder was divided into nine 500 g portions. The nine alkaline treatment combinations were randomly assigned to these nine FFBR powder portions. The nine alkaline treatment combinations were heated to 90 °C. Then, the FFBR powder was added to the solution, stirred to well mix, and immediately placed in a hot water bath (90 °C). After alkaline treatment, the FFBR samples were removed from the water bath, the alkaline solution was drained, and the samples were neutralized by washing with tap water until the pH of the water became neutral. Treated FFBR powder was dried in a hot air oven at 100 °C until its water activity reached ≤0.6. This dried FFBR powder, called biocalcium, was then subjected to analysis of the chemical composition (crude protein, crude fat, ash content, calcium, phosphorus, crude fiber, and salt content), CIE color values, morphological structure, and the intensity of the fermented fish smell.

#### 2.1.3. Biocalcium Analyses

Crude protein was determined using the Kjeldahl method according to AOAC method 990.03 [27].

Crude fat was determined based on a petroleum ether extraction method using the Soxhlet apparatus (Soxtherm, model SOX416, C. Gerhardt GmbH & Co. KG, Königswinter, Germany) according to AOAC method 954.02 [27].

Ash content was determined according to AOAC method 942.05 [27]. Two grams of biocalcium were placed in a crucible. Then, the sample was incinerated in a muffle furnace at 600 °C for 4 h (Automatic Muffle Furnace MFP-300N, IKEDA Scientific Co., Ltd., Tokyo, Japan), cooled in a desiccator, and weighed.

Calcium and phosphorus contents were determined using atomic absorption spectroscopy (AAS) and spectrophotometry, respectively. Each sample was digested using the wet digestion method with nitric and perchloric acids, according to Uddin et al. [28]. Calcium content was determined using a contrAA 800 atomic absorption spectrometer (Analytik Jena, Überlingen, Germany) at 422 nm. Phosphorus content relying on color development reaction with molybdovanadate was determined using a SPECORD 210 PLUS spectrophotometer (Analytik Jena, Überlingen, Germany) at 420 nm.

The crude fiber was determined using AOAC method 962.09 [27]. Briefly, 1 g of the sample was placed in a FibreBag in the Fiber Apparatus (Gerhart Fibretherm, C. Gerhart GmbH & Co. KG, Königswinter, Germany). Then, 1.25% sulfuric acid and 1.25% NaOH was added. After extraction, the sample was washed with distilled water, dried at 100 °C overnight, incinerated in a muffle furnace at 600 °C for 4 h (Automatic Muffle Furnace MFP-300N, IKEDA Scientific Co., Ltd.), cooled in a desiccator, and weighed.

The salt content was determined according to the AOAC volumetric method (937.09) [27]. Briefly, 0.5 g of sample was mixed with 20 mL of standardized 0.1 N silver nitrate and 20 mL of 65% nitric acid in a 250-mL Erlenmeyer flask. The mixture was boiled on a hot plate for 15 min to decompose the sample, except for silver chloride particles, cooled to ambient temperature, and mixed with 50 mL of distilled water and 5 mL of saturated ferric indicator. Then, the mixture was titrated with standardized 0.1 N ammonium thiosulfate until it became permanently brown. The amount of 0.1 N ammonium thiosulfate was subtracted from the amount of 0.1 N silver nitrate added. The difference in volume of these two standardized solutions was used to calculate the sodium chloride content (%). Note that 1 g of sample for each 1 mL of 0.1 N silver nitrate is equivalent to 0.0058% sodium chloride.

CIE color values (L*, a*, and b*) were measured with a ColorFlex EZ Spectrophotometer (Hunter Associates Laboratory, Inc., Reston, VA, USA). The biocalcium sample was illuminated with a D65 artificial daylight bulb and observed with a 10° standard angle observer. L* represents lightness on a vertical axis with scale values from 0 (black) to 100 (white). a* and b* indicate red (+a*)/green (−a*) and yellow (+b*)/blue (−b*) coordinates, respectively. a* and b* have no numeric limits.

The morphological structure of washed FFBR and biocalcium samples was observed using a scanning electron microscope (S-800, Hitachi Ltd., Tokyo, Japan) at an accelerating voltage of 15 kV. Each sample was mounted with the uppermost inner slides on aluminum stubs, coated with platinum using a vacuum coater (RMC-Eiko RE, Eiki Engineering Co., Ltd., Ibaraki, Japan), and observed directly at 1000× magnification.

The intensity of the fermented fish smell in the biocalcium samples was evaluated using the odor intensity scale (0 = not perceptible, 1 = very weak, 2 = weak, 3 = distinct, 4 = strong, 5 = very strong, and 6 = extremely strong) [29]. Prior to evaluation, 20 g of the biocalcium sample was placed in a 100 mL squeezable plastic bottle with a narrow tip. The bottle was capped tightly, given a randomly assigned three-digit number, and then presented to 45 panelists who were acquainted with fermented fish/fish smell and not allergic to it. The panel comprised 24 women and 21 men aged 19–45 years. Sensory evaluation was carried out in an air-conditioned room at 25 °C. During evaluation, the panelists uncapped and gently squeezed the bottles, then immediately sniffed the tips of the bottles and recorded the intensity of the fermented fish smell.

### 2.2. Fortification of FES with the Properly Treated Biocalcium and Investigation of the Effects of Biocalcium Fortification on FES Quality Parameters

#### 2.2.1. FES Fortification with Biocalcium

**Figure 1:**
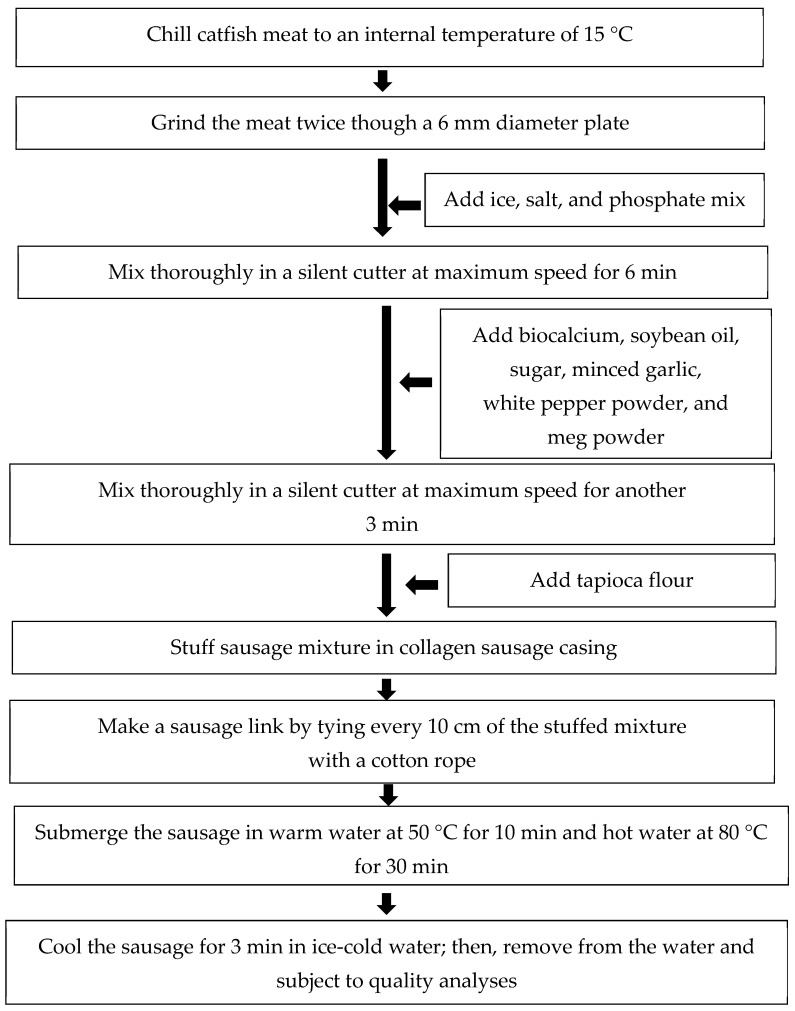
Preparation of fish emulsion sausage.

Biocalcium powder was prepared according to Section 2.1.2. The most suitable alkaline treatment was used based on the first experiment. The powder was sieved using a stainer with a 90 μm sieve and fortified in FES. The recipe used to prepare FES was modified from Tirasarot and Wongtanon [30]; it is shown in Table 1. Figure 1 shows the steps for FES preparation [2].

#### 2.2.2. Quality Analyses of FES

Calcium, phosphorus, crude fiber, and salt contents in FES were measured as described in Section 2.1.3.

pH was determined according to Jung et al. [31]. Ten grams of ground FES was combined with 100 mL of recently boiled deionized water. The mixture was homogenized for 20 s, and the pH was measured with a PP150 desktop pH meter (Sartorius Corp., Edgewood, NY, USA).

Internal color values (L*, a*, and b*) of cut FES were measured using a ColorFlex EZ Spectrophotometer (Hunter Associates Laboratory, Inc.) as described in Section 2.1.3.

Texture profile analysis (TPA) was performed with a texture analyzer (TA-XT2i, Stable Micro Systems Ltd., Vienna, Austria) equipped with a 36 mm cylinder probe. Each FES sample was cut into a cylinder (height of 25 mm). It was measured at a crosshead speed of 5 mm/s and compressed twice to 75% of its original height [18]. Six cylinder-shaped samples obtained from each treatment were subjected to TPA analyses. TPA provided the hardness (kg), adhesiveness (g.s), springiness, cohesiveness, gumminess (kg), and chewiness (kg) of each sample.

Emulsion stability was determined according to Jeong and Han [32]. A 20 g sausage batter was gently placed to a centrifuge tube to avoid bubbles in the sausage matrix and the tube was submerged in a water bath at 80 °C for 30 min. After 20 min incubation at room temperature, the volumes of water and fat collected in the centrifuge tube were measured separately. The water and fat losses were expressed as percentages of the batter weight according to Equations (1) and (2), respectively:

(1) $$Water\;loss\left(\%\right)=water\;layer\left(mL\right)/sausage\;batter\;weight\left(g\right)\times 100$$

(2) $$Fat\;loss\left(\%\right)=fat\;layer\left(mL\right)/sausage\;batter\;weight(g)\times 100$$

Sensory acceptability (interior appearance, interior color, odor, mouthfeel texture, flavor, and overall acceptability) and the intensity of the fermented fish smell of the FES samples were evaluated in an air-conditioned room at 25 °C by 45 panelists familiar with FES and not allergic to it. The panel comprised 23 women and 22 men aged 19–45 years. The sensory acceptability was determined by using a 9-point hedonic scale according to Meilgaard et al. [33] (1 = dislike extremely, 5 = neither like nor dislike, 9 = like extremely). A score of 5 was considered the cut-off point for all sensory acceptability attributes. The same panelists rated the intensity of the fermented fish smell by using the odor intensity scale described in Section 2.1.3.

### 2.3. Statistical Analysis

Exploring the optimal alkaline treatment in the first experiment was performed using a 3 × 3 factorial arrangement in a randomized complete block design (RCBD). The experiment was repeated three times using FFBR from three different processing lots. The investigated factors were the NaOH concentration (0%, 3%, and 6%) and the soaking time (0, 1, and 2 h). The data were subjected to a two-way analysis of variance (ANOVA) using SPSS Statistics 21 (IBM Corp., Armonk, NY, USA). Biocalcium fortification in FES in the second experiment was performed using an RCBD. The experiment was repeated three times using biocalcium from three different processing lots. The investigated factor was the amount of biocalcium (0, 12, 24, and 36 g) used to fortify FES. The data were subjected to one-way ANOVA using the statistical program, as previously mentioned. Differences between the mean values were established using the least significant difference (LSD) at a 95% confidence level.

## 3. Results and Discussion

### 3.1. Investigation of the NaOH Concentration and Soaking Time That Yield the Highest Quality Biocalcium from FFBR

#### 3.1.1. Crude Protein

Protein is considered an impurity in biocalcium. Prabu et al. [34] have reported that NaOH can effectively dissolve proteinaceous substances. Likewise, multiple studies have reported the removal of protein in fish bone and biocalcium using alkaline (NaOH or KOH) treatments and soaking times [5,8,35,36,37,38,39].

**Figure 2:**
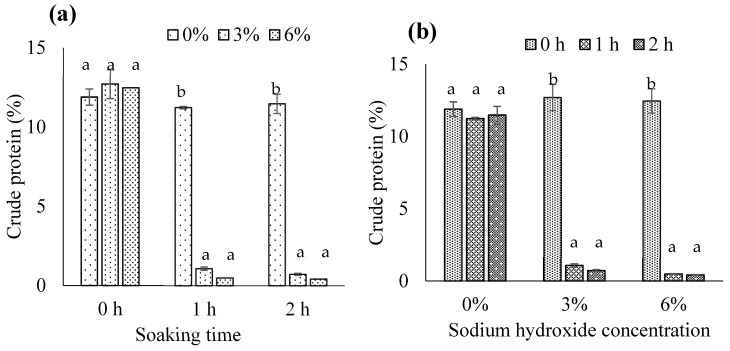
Crude protein in biocalcium produced from fermented fish bone residue based on (**a**) the sodium hydroxide concentration and (**b**) the soaking time. Different letters above the bars for each soaking time or sodium hydroxide concentration indicate significant differences (*p* < 0.05) (*n* = 3).

We found a significant NaOH concentration × soaking time interaction for crude protein (*p* < 0.05). Consistent with Nemati et al. [5], when the soaking time was 1 or 2 h, 3% or 6% NaOH significantly reduced crude protein in biocalcium compared with 0% NaOH (*p* < 0.05) (Figure 2a). Alkaline was able to solubilize the protein attached to fish bone and lead to the removal of proteinaceous substances from the bones [35]. During exposure to alkaline treatment at elevated temperatures, proteins were likely denatured or unfolded, thus increasing in solubilization of denatured proteins and subsequently causing mass diffusivity/transfer from the bone matrix [35]. Therefore, alkaline treatment could decrease crude protein in experimental biocalcium.

We identified a 3% NaOH treatment and soaking for 1 h as the most suitable to prepare biocalcium from FFBR, reducing crude protein from 12–13% to around 1% (Figure 2b). This condition would reduce resource investment compared to longer and higher concentration NaOH treatments. Optimal alkaline treatment may be variable depending on the nature of fish bones and any pretreatments (e.g., high-pressure water jet cleaning [35]). We recommend that biocalcium producers use pre-alkaline treatments to minimize protein on fish bones, followed by NaOH soaking at a concentration and soaking time suitable for their materials. 

#### 3.1.2. Crude Fat

**Figure 3:**
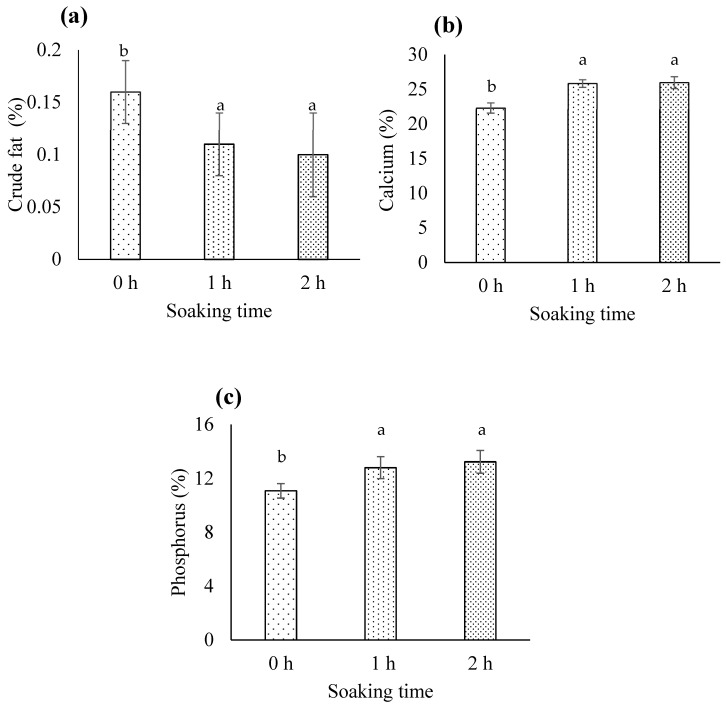
The (**a**) crude fat, (**b**) calcium, and (**c**) phosphorus contents in biocalcium for each soaking time. Different letters above the bars for each parameter indicate significant differences (*p* < 0.05) (*n* = 9).

Fat can induce oxidative rancidity in biocalcium derived from FFBR, necessitating its removal. NaOH effectively saponifies fats, allowing for their emulsification and subsequent removal during alkaline treatment [34]. We observed a non-significant NaOH concentration × soaking time interaction for crude fat (*p* > 0.05). The tested NaOH concentrations did not significantly impact crude fat levels in biocalcium (*p* > 0.05). However, soaking time did influence crude fat content. Extending soaking time from 0 to 1 or 2 h significantly reduced crude fat from 0.16% to approximately 0.1% (*p* < 0.05; Figure 3a), with no significant difference observed between 1 and 2 h (*p* > 0.05). These findings suggest that a soaking time of 1 h is optimal, irrespective of NaOH concentration. Nemati et al. [5] and Amitha et al. [8] noted varying fat contents (7.26–11.02%) in fish bones from different species, with exposure to 2% NaOH for 30 min reducing fat content to <3.86%.

Notably, FFBR exhibited low-fat content (0.16%), and application of this optimal condition could achieve a 37.5% reduction in crude fat (from 0.16% to 0.1%; Figure 3a). This reduction may result from the saponification and emulsification processes previously described. Under appropriate storage conditions, such as vacuum packaging, any residual fat may not adversely affect biocalcium quality.

#### 3.1.3. Ash Content

**Figure 4:**
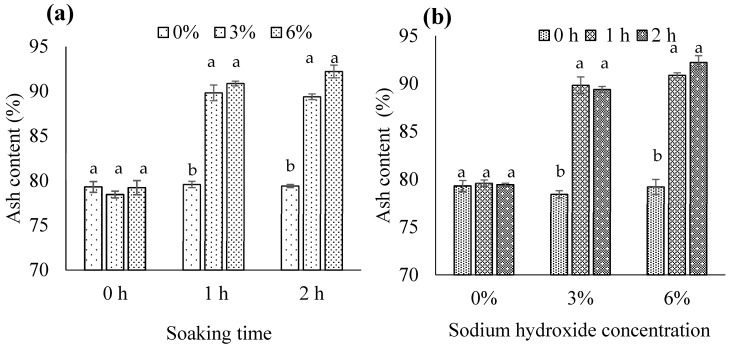
Ash content in biocalcium produced from fermented fish bone residue based on (**a**) the sodium hydroxide concentration and (**b**) the soaking time. Different letters above the bars for each soaking time or sodium hydroxide concentration indicate significant differences (*p* < 0.05) (*n* = 3).

Ash content denotes the total inorganic minerals present in food [40], with higher ash content in biocalcium indicating the removal of more unwanted organic material. We observed a significant NaOH concentration × soaking time interaction for ash content (*p* < 0.05). Specifically, when soaking time was 1 or 2 h, ash content significantly increased with alkaline treatment using 3% or 6% NaOH compared to 0% NaOH (*p* < 0.05), with no distinction between the 3% and 6% NaOH concentrations (Figure 4a). Additionally, when utilizing 3% or 6% NaOH, a soaking time of 1 or 2 h significantly elevated ash content compared to 0 h (*p* < 0.05), with no discrepancy between the 1 and 2 h soaking times (Figure 4b). The removal of proteinaceous impurities from biocalcium by alkaline treatment may elevate the ratio of insoluble inorganic minerals, thereby enhancing ash content. These findings align with the existing literature [5,8,36,39]. Accordingly, we identified 3% NaOH and a 1 h soaking time as the most suitable alkaline treatment. 

#### 3.1.4. Calcium

**Figure 5:**
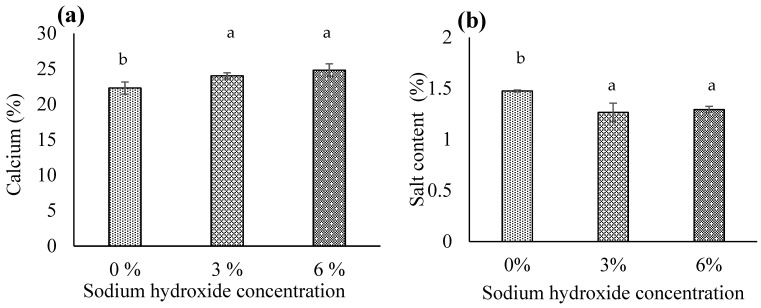
The (**a**) calcium content and (**b**) salt content based on the sodium hydroxide concentration. Different letters above the bars for each parameter indicate significant differences (*p* < 0.05) (*n* = 9).

The NaOH concentration × soaking time interaction was not significant (*p* > 0.05). However, both NaOH concentration and soaking time individually significantly affected calcium content in biocalcium (*p* < 0.05). Specifically, 3% or 6% NaOH significantly increased calcium content compared to 0% NaOH (*p* < 0.05), with no significant difference between 3% and 6% NaOH concentrations (Figure 5a). Additionally, soaking for 1 or 2 h significantly increased calcium compared to 0 h (*p* < 0.05), with no significant difference between 1 and 2 h (Figure 3b). Thus, 3% NaOH with a 1 h soak is preferable to other conditions. Higher NaOH concentrations and longer soaking times can remove impurities like fish meat, increasing the ash content in biocalcium. Since calcium is a component of ash, this rise in ash content can directly elevate the calcium levels in the product. These findings align with Benjakul et al. [37]. The optimal treatment condition in our study (3% NaOH with a 1 h soak) yielded 24.78% calcium in biocalcium, surpassing calcium content in biocalcium from tuna bone (19–22%) [37,38]. 

#### 3.1.5. Phosphorus

Phosphorus is the second most abundant mineral in fish bone after calcium [41]. There was no significant interaction between NaOH concentration and soaking time for phosphorus (*p* > 0.05). While NaOH concentration had no effect on phosphorus content (*p* > 0.05), soaking time did (*p* < 0.05). Soaking for 1 or 2 h significantly increased phosphorus content compared to 0 h (*p* < 0.05), with no difference between 1 and 2 h (Figure 3c). Regardless of NaOH concentration, soaking for 1 h is more efficient than for 2 h. This duration could raise phosphorus content from 11.08% to 13.23%. The increase in phosphorus content observed here exceeds that reported by Benjakul et al. [37], who found that alkaline treatment with 2 M NaOH and 30 min of soaking increased phosphorus in biocalcium-produced tuna bone from 9.51% to 10.02%. Increasing ash content following fish meat removal via alkaline treatment may subsequently raise phosphorus levels, a mineral found in ash.

#### 3.1.6. Crude Fiber

The crude fiber in biocalcium may come from roasted rice/rice bran added to the fermented fish. However, a low crude fiber content is preferred as it is considered an impurity. We observed no significant interaction between NaOH concentration and soaking time for crude fiber content (*p* > 0.05), indicating that neither factor affected it (*p* > 0.05). This contrasts with Mahdi et al. [42], who found that alkaline treatment could remove fiber from foods. It is possible that our NaOH concentrations were too low to disintegrate crude fiber in biocalcium. Given the already low crude fiber content in our samples (0.27–0.89%), using higher NaOH concentrations (>6%) to remove fiber may not be beneficial. 

#### 3.1.7. Salt Content

There was no significant interaction between NaOH concentration and soaking time for salt content (*p* > 0.05). However, NaOH concentration influenced salt content in biocalcium (*p* < 0.05). Specifically, treatment with 3% or 6% NaOH significantly reduced salt content compared to 0% NaOH (*p* < 0.05), with no significant difference between 3% and 6% NaOH concentrations (Figure 5b). Salt may be present in fish meat found in FFBR, and alkaline treatment removes the meat and thus salt. Treating FFBR with 3% or 6% NaOH could reduce salt content from 1.48% to 1.27–1.29%. Soaking time did not significantly affect salt content (*p* > 0.05). Using 3% NaOH would be suitable for salt removal regardless of soaking time, conserving resources.

#### 3.1.8. CIE Color Values

**Figure 6:**
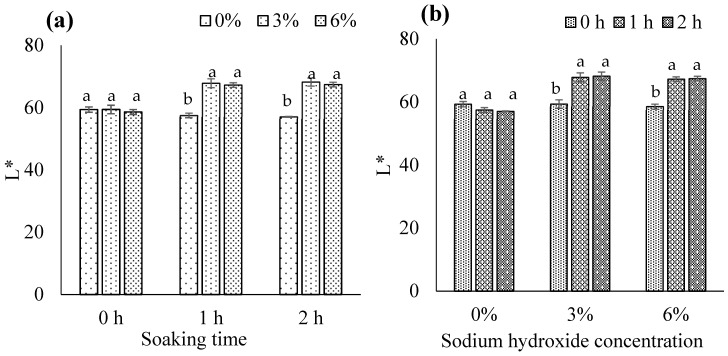
L* of biocalcium produced from fermented fish bone residue based on (**a**) the sodium hydroxide concentration and (**b**) the soaking time. Different letters above the bars for each soaking time or sodium hydroxide concentration indicate significant differences (*p* < 0.05) (*n* = 3).

The NaOH concentration × soaking time interaction was significant for L* (*p* < 0.05). With a soaking time of 1 or 2 h, biocalcium exposed to 3% or 6% NaOH showed significantly higher L* compared to biocalcium exposed to 0% NaOH (*p* < 0.05; Figure 6a). When using 3% or 6% NaOH, L* significantly increased with soaking time from 0 h to 1 or 2 h (*p* < 0.05; Figure 6b). Fermented fish meat in FFBR typically has a brown color due to Maillard reactions during long fermentation (>6 months) [2]. Increased removal of brown fermented fish meat in biocalcium with higher NaOH concentration and longer soaking time may lead to an increase in L*. Niwet et al. [36] and Benjakul et al. [37] also reported an increase in L* of fish biocalcium after alkaline treatment. Since there were no significant differences between 3% or 6% NaOH and soaking for 1 or 2 h, using 3% NaOH and soaking for 1 h is the most suitable condition for producing biocalcium from FFBR. 

**Figure 7:**
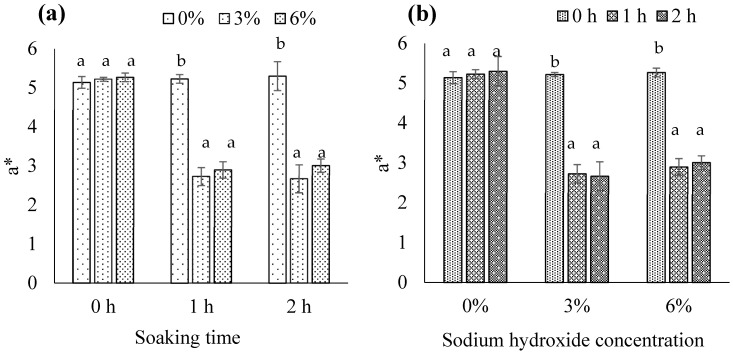

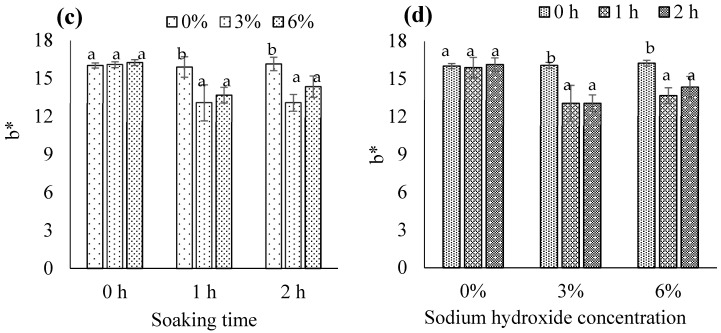
a* and b* of biocalcium produced from fermented fish bone residue based on (**a**,**c**) the sodium hydroxide concentration and (**b**,**d**) the soaking time. Different letters above the bars for each soaking time or sodium hydroxide concentration indicate significant differences (*p* < 0.05) (*n* = 3).

The NaOH concentration × soaking time interaction was also significant for a* and b* (*p* < 0.05). With a soaking time of 1 or 2 h, 3% or 6% NaOH significantly reduced a* and b* (*p* < 0.05); there was no difference between 3% and 6% NaOH (Figure 7a,c). A soaking time of 1 or 2 h significantly reduced a* and b* when using 3% or 6% NaOH (*p* < 0.05); there was no difference between 1 and 2 h (Figure 7b,d). Removal of brown fermented fish meat in biocalcium by alkaline treatment as previously described may contribute to the reduction in a* and b*. The results agree with Benjakul et al. [37], who found that alkaline treatment could reduce a* and b* of biocalcium produced from tuna bone. Based on the results, 3% NaOH and soaking for 1 h is most suitable for treating FFBR to obtain biocalcium. 

**Figure 8:**
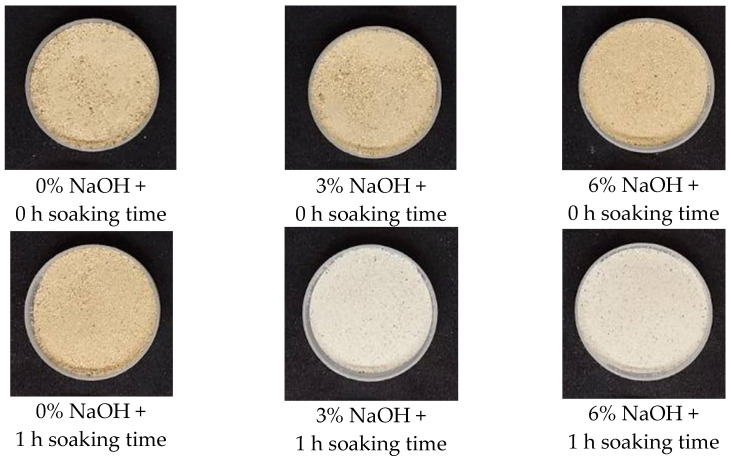

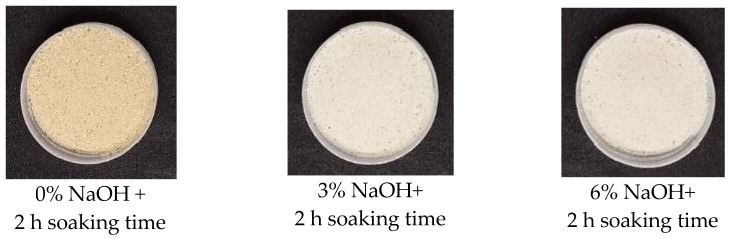
The color of biocalcium powder obtained from different treatments.

A reduction in the color intensity plays an important role in biocalcium quality. As shown in Figure 8, both biocalcium samples, which were exposed to 0% NaOH (the first column on the left) or 0 h soaking time (the first row on the top), appeared brown. However, biocalcium treated with 3% or 6% NaOH and soaked for 1 or 2 h appeared off-white. We found that 3% NaOH and soaking for 1 h, 3% NaOH and soaking for 2 h, 6% NaOH and soaking for 1 h, and 6% NaOH and soaking for 2 h produced the best color. To conserve resources, 3% NaOH and soaking for 1 h should be used to treat FFBR to obtain biocalcium. 

#### 3.1.9. Morphological Structure of Biocalcium Powder

**Figure 9:**
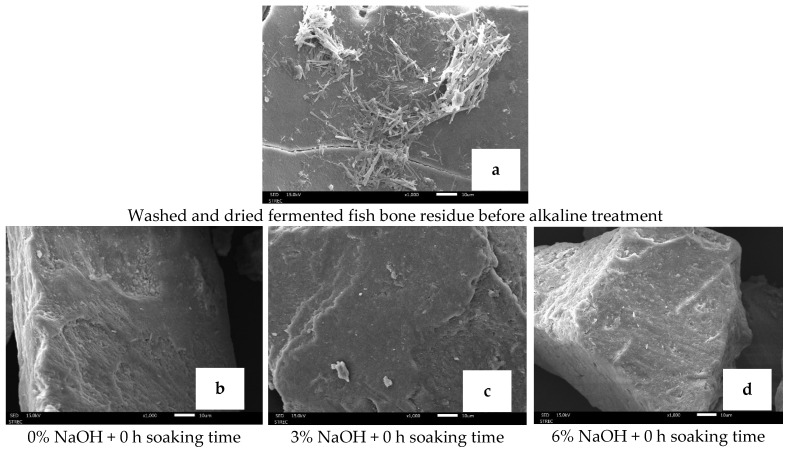

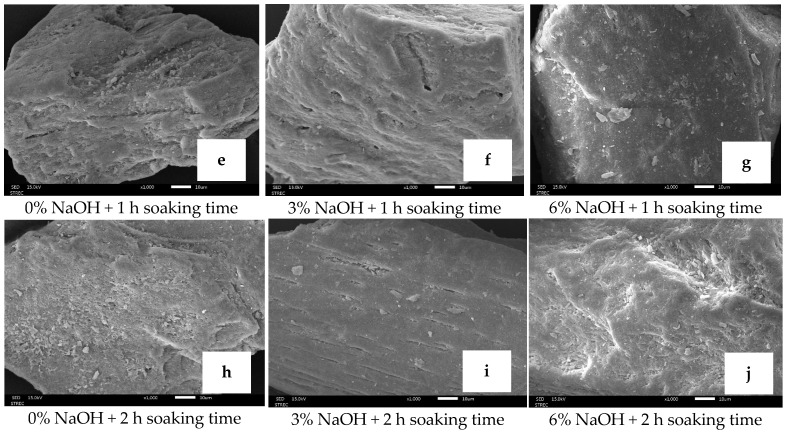
Morphological structure at 1000× magnification and 15 kV acceleration of (**a**) fermented fish bone residue after being washed, autoclaved, and dried and (**b**–**j**) biocalcium particles obtained after different alkaline treatments.

After washing, autoclaving, and drying, FFBR still retained fish muscle fiber on its surface (Figure 9a). Pulverization of FFBR may have separated this muscle from the bone, breaking it into particles. The solution used for biocalcium treatment can alter its morphological characteristics. For instance, Wang [43] noted erosion on biocalcium surfaces exposed to acetic acid. However, various NaOH concentrations and soaking times did not change the surface morphology of biocalcium particles (Figure 9b–j). Alkaline treatment with 3% or 6% NaOH and soaking for 1 or 2 h did not corrode the surface of biocalcium particles (Figure 9f,g,i,j), thus ensuring the retention of essential minerals like calcium and phosphorus. These findings differ from Techochatchawal et al. [44], who observed erosion on biocalcium produced from Nile tilapia bone treated with alkaline (0.8% NaOH, 1 h soak) and subjected to heat treatment under pressure, suggesting caution against using high pressures during alkaline treatment.

#### 3.1.10. Intensity of the Fermented Fish Smell in Biocalcium Powder

**Figure 10:**
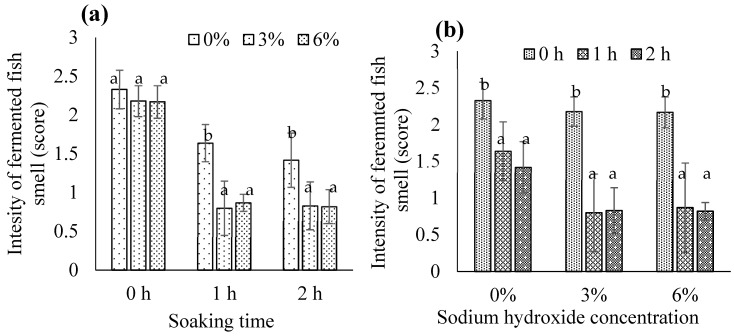
The intensity of the fermented fish smell in biocalcium produced from fermented fish bone residue based on (**a**) the sodium hydroxide concentration and (**b**) the soaking time. Different letters above the bars for each soaking time or sodium hydroxide concentration indicate significant differences (*p* < 0.05) (*n* = 3).

There was a significant interaction between NaOH concentration and soaking time for the intensity of the fermented fish smell (*p* < 0.05) (Figure 10). Both 3% and 6% NaOH, with 1 or 2 h of soaking, significantly reduced the smell compared to 0% NaOH (*p* < 0.05); no difference was observed between 3% and 6% NaOH (Figure 10a). This suggests that soaking for 1 or 2 h with 3% NaOH is the most effective treatment for reducing the fermented fish smell in biocalcium. Regardless of the NaOH concentration, soaking for 1 or 2 h significantly reduced the intensity of the fermented fish smell compared with 0 h (*p* < 0.05); there was no difference between 1 and 2 h (Figure 10b). To conserve resources, soaking for 1 h with 3% NaOH should be used to treat FFBR to obtain biocalcium. Either dissolution of smelly fermented fish meat due to alkaline exposure or leaching of the fermented fish smell during soaking may contribute to the less intense fermented fish smell of biocalcium.

For optimal biocalcium production from FFBR, 3% NaOH with 1 h soaking is ideal, resulting in a 24.78% calcium and 13.11% phosphorus content, alongside effective protein removal, ash improvement, color change, reduced fish smell intensity, and minimized resource investment. The calcium-to-phosphorus ratio of this experimental biocalcium (1.89) is higher than those reported by Yin et al. [45] and Boutinguiza et al. [46] (1.53 and 1.67, respectively).

### 3.2. Fortification of FES with the Properly Treated Biocalcium and Investigation of the Effects of Biocalcium Fortification on FES Quality Parameters

#### 3.2.1. Calcium

Biocalcium, which is generally derived from fish bones, is an excellent source of calcium and phosphorus [47]. In our study, unfortified FES contained only 1.33 mg of calcium per 100 g (Table 2). Fortification with biocalcium at 12, 24, or 36 g increased calcium levels to 56.93, 108.67, and 163.33 mg/100 g, respectively (Table 2), enhancing the calcium content by up to 123-fold, which aligns with the findings of prior studies [18,19,20,24]. 

Thai Food and Drug Administration [48] permits calcium fortification up to 400 mg per serving. Emulsion sausages sold in convenience shops in Thailand mostly weight around 150 g per serving. Our fortified FES products, at 85.40, 163.00, and 245.00 mg of calcium per 150 g serving, comply with this regulation. According to the Ministry of Public Health of Thailand [49], labels can claim “good source of calcium” and “high calcium” for products that are 10–19% (80–152 mg per serving) and ≥20% (≥160 mg per serving) of Thai recommended daily intakes/serving (800 mg calcium), respectively. The addition of 12 g or 24 g of biocalcium would allow the sausage manufacturers to claim “good source of calcium” and “high calcium” on the product labels, respectively. Therefore, FES with 12 g or 24 g of biocalcium qualifies for these nutritional claims, potentially influencing consumer choices.

#### 3.2.2. Phosphorus

The phosphorus content increased significantly as the amount of added biocalcium increased (*p* < 0.05; Table 2). Of note, the phosphorus content in unfortified FES (0 g) was as high as 259.33 mg/100 g. This phosphorus may originate from the phosphate mix added during preparation. The results agree with the study by Wijayanti et al. [22], who found that the addition of biocalcium powder in surimi could increase the phosphorus content in the product.

#### 3.2.3. Crude Fiber and Salt Contents

The crude fiber and salt contents were not significantly different among the FES fortified with amounts of biocalcium (*p* > 0.05; Table 2). The low crude fiber (0.27–0.89%) and salt (1.27–1.29%) contents in biocalcium likely underlie the lack of an effect on the crude fiber and salt contents in fortified FES.

#### 3.2.4. pH

The addition of biocalcium did not significantly affect the pH of FES (*p* > 0.05; Table 3). After the alkaline treatment of biocalcium (3% NaOH and soaking for 1 h), it was neutralized by washing with water. Hence, the addition of neutralized biocalcium to FES did not affect its pH. This result agrees with Hemung and Sriuttha [19], who discovered that the pH of FES did not change due to fortification with fish bone extract.

#### 3.2.5. CIE Color Values

**Figure 11:**
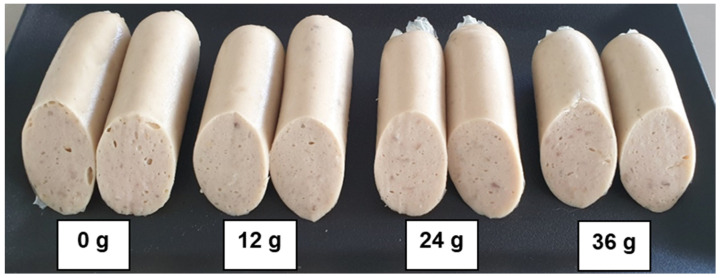
The appearance of fish emulsion sausage fortified with 0, 12, 24, or 36 g of biocalcium produced from fermented fish bone residue.

Adding 12, 24, or 36 g of biocalcium to FES did not significantly alter its color (*p* > 0.05; Table 3; Figure 11), despite biocalcium’s light color (L* 68.57, a* 2.51, b* 13.65). This minimal use of biocalcium did not impact FES’s color metrics significantly. This finding contrasts with studies by Hemung and Sriuttha [19], Yin et al. [21], and Wijayanti et al. [22], who reported that biocalcium could increase the L* value in food products. However, our results align with Hemung and Sriuttha [19] regarding the unchanged a* and b* values, while Wijayanti et al. [22,23] and Pudtikajorn et al. [24] observed increases in a* and b* values in fish-based gel products through biocalcium fortification.

#### 3.2.6. Texture Profile Analysis

Biocalcium fortification did not significantly affect the hardness and springiness of FES (*p* > 0.05) (Table 3). However, it significantly influenced adhesiveness, cohesiveness, gumminess, and chewiness (*p* < 0.05), with unfortified FES being more adhesive. Adhesiveness is defined as the work necessary to overcome the attractive force between the surface of the food and the surface of other materials with which the food comes into con-tact [50]. Nanoscale biocalcium power has been shown to improve the texture properties of surimi [51]. The particle size of biocalcium, around 90 μm in this study, is suggested to affect these texture properties, indicating that smaller particles may enhance FES’s textural qualities. 

Cohesiveness indicates the strength of internal bonds making up the body of food [52]. Cohesiveness was reduced in FES fortified with biocalcium, possibly due to the disruption of internal bonds by the larger particle size. Gumminess is defined as the energy required to disintegrate a semisolid food product to a state ready for swallowing [50]. Differently, chewiness is referred to the energy required to masticate a solid [50]. Similarly, FES with biocalcium was less gummy and chewy. The biocalcium particle size used in this experiment may have influenced the gumminess and the chewiness. Our findings contrast with Hemung et al. [18,19] and Wijayanti et al. [22,23] but align with Bhenjapaipong et al. [25], who noted that larger biocalcium particles decreased cohesiveness in fish balls. Future studies should explore the impact of smaller biocalcium particles on FES’s texture.

#### 3.2.7. Emulsion Stability

Emulsion stability is the ability to maintain moisture, fat, and added moisture, which the meat originally had during the process of cutting and heating [32]. Biocalcium fortification did not significantly affect the water and fat losses of FES indicating emulsion stability (*p* > 0.05; Table 3). The results suggest that addition of biocalcium with particle size of 90 µm in FES did not alter its emulsion stability. Emulsion stability of emulsion sausages can be maintained by addition of phosphate derivatives; thus recommending for FES production.

#### 3.2.8. Sensory Evaluation

Unlike some textural properties, fortification with biocalcium did not significantly affect the FES sensory attributes (*p* > 0.05; Table 4). The amount of biocalcium used to fortify fish-based gel products may play an important role in the acceptability of the products. The low levels of biocalcium used in this experiment likely underlie the lack of an effect on the sensory acceptability of the product.

Although the experimental biocalcium had been subjected to a suitable alkaline treatment (3% NaOH and soaking for 1 h), it still had a mild fermented fish smell (0.8 points on the 6-point scale; Figure 10). After fortification with biocalcium, the panelists did not detect a fermented fish smell in FES. The small amount of biocalcium added to FES may produce no or a very low fermented fish smell. Moreover, the other ingredients in the product, particularly spices, could mask this mild smell. Our findings disagree with the study by Pudtikajorn et al. [24], who found that the acceptability score of fish tofu decreased as the level of biocalcium increased.

## 4. Conclusions

We compared different NaOH concentrations (0%, 3%, and 6%) and soaking times (0, 1, and 2 h) to determine the most suitable alkaline treatment conditions to produce biocalcium from FFBR. We found that 3% or 6% NaOH was better than 0% NaOH regarding crude protein, ash content, calcium, CIE color values, salt content, and intensity of the fermented fish smell. Additionally, a soaking time of 1 or 2 h yielded better results compared with 0 h in terms of crude protein, ash content, calcium, phosphorus, crude fat, CIE color values, and intensity of the fermented fish smell. The 3% NaOH and soaking for 1 h, 3% NaOH and soaking for 2 h, 6% NaOH and soaking for 1 h, and 6% NaOH and soaking for 2 h treatments were not significantly different. Based on resource conservation, 3% NaOH and soaking for 1 h is the most suitable alkaline treatment condition.

We found that fortifying FES with biocalcium obtained from FFBR subjected to the ideal alkaline treatment could improve the calcium and phosphorus contents. Fortification with biocalcium had minor effects on the adhesiveness, cohesiveness, gumminess, and chewiness of FES. However, most of the quality parameters of FES–including pH, CIE color values, crude fiber, salt content, hardness, springiness, emulsion stability, and sensory evaluation scores–were not affected by biocalcium fortification. Therefore, it is possible to fortify FES by adding 12, 24, or 36 g of biocalcium generated from FFBR. The amount of biocalcium that should be added to FES depends on the label claim the FES producer would like to make. The addition of 12 and 24 g of biocalcium would enable the producers to claim “good source of calcium” and “high calcium”, respectively, on FES labels. The results suggest that a biocalcium particle size of 90 μm may slightly decrease the adhesiveness, cohesiveness, gumminess, and chewiness of the FES; thus, using biocalcium particles < 90 μm should be explored in the future. In addition, the storage stability of FES and its shelf-life under air and vacuum packaging should be investigated. Our study is an example of utilizing a local fish by-product to produce value-added products. We hope these findings inspire researchers outside of Thailand to explore the use of such by-products.

## Figures and Tables

**Table 1:** Fish emulsion sausage composition.

Composition (g)	Treatment			
	0	12	24	36
Ground catfish meat	5000	4988	4976	4964
Biocalcium	0	12	24	36
Tapioca flour	265	265	265	265
Soybean oil	125	125	125	125
Ice	1000	1000	1000	1000
Phosphate mix *	25	25	25	25
Salt	100	100	100	100
Sugar	35	35	35	35
Minced garlic	138	138	138	138
White pepper powder	25	25	25	25
Meg powder	8	8	8	8
Total weight	6721	6721	6721	6721

* Tetrasodium diphosphate 60%, disodium diphosphate 25%, and sodium polyphosphate 15%.

**Table 2:** Calcium, phosphorus, crude fiber, and salt content in fish emulsion sausage fortified with biocalcium.

Quality Parameter	Level of Biocalcium (g)			
	0	12	24	36
Calcium (mg/100 g)	1.33 ± 0.52d	56.93 ± 4.15c	108.67 ± 2.33b	163.33 ± 3.46a
Phosphorus (mg/100 g)	259.33 ± 2.81d	279.23 ± 2.55c	316.02 ± 3.60b	343.87 ± 3.06a
Crude fiber (%)	0.83 ± 0.36a	0.84 ± 0.23a	0.85 ± 0.11a	0.85 ± 0.26a
Salt content (%)	1.50 ± 0.53a	1.53 ± 0.62a	1.51 ± 0.79a	1.56 ± 0.67a

The data are presented as the mean ± standard deviation. Different letters in the same row indicate significant differences (*p* < 0.05).

**Table 3:** pH, CIE color values, and texture profile analysis of fish emulsion sausage fortified with biocalcium.

Quality Parameter		Level of Biocalcium (g)			
		0	12	24	36
pH		6.71 ± 0.13a	6.74 ± 0.28a	6.70 ± 0.25a	6.77 ± 0.26a
CIE color values	L*	64.28 ± 2.59a	65.68 ± 1.43a	66.24 ± 1.11a	66.70 ± 1.14a
	a*	1.38 ± 0.39a	1.23 ± 0.46a	1.01 ± 0.09a	1.37 ± 0.48a
	b*	17.82 ± 0.55a	17.92 ± 0.74a	18.53 ± 0.26a	18.49 ± 0.86a
Texture profile analysis	Hardness (kg)	8.99 ± 0.23a	8.56 ± 0.21a	8.82 ± 0.15a	8.66 ± 0.20a
	Adhesiveness (g.s)	−27.71 ± 0.89a	−24.23 ± 0.22b	−24.16 ± 0.10b	−24.88 ± 0.35b
	Springiness	0.84 ± 0.02a	0.84 ± 0.02a	0.84 ± 0.02a	0.84 ± 0.03a
	Cohesiveness	0.52 ± 0.05a	0.40 ± 0.06b	0.40 ± 0.07b	0.42 ± 0.07b
	Gumminess (kg)	4.75 ± 0.13a	3.40 ± 0.10b	3.48 ± 0.10b	3.58 ± 0.16b
	Chewiness (kg)	4.41 ± 0.58a	3.55 ± 0.62b	3.52 ± 0.74b	3.33 ± 0.36b
Emulsion stability	Water loss (%)	13.65 ± 0.81a	14.78 ± 0.62a	14.53 ± 0.52a	14.87 ± 0.82a
	Fat Loss (%)	4.82 ± 0.74a	5.13 ± 0.68a	5.26 ± 0.73a	5.34 ± 0.67a

The data are presented as the mean ± standard deviation. Different letters in the same row indicate significant differences (*p* < 0.05).

**Table 4:** Sensory evaluation scores of fish emulsion sausage fortified with biocalcium.

Quality Parameter		Level of Biocalcium (g)			
		0	12	24	36
Sensory evaluation (score)	Interior appearance	6.70 ± 1.40a	6.75 ± 1.60a	6.82 ± 1.57a	6.72 ± 1.70a
	Interior color	6.62 ± 1.55a	6.46 ± 1.56a	6.66 ± 1.49a	6.38 ± 1.72a
	Odor	6.36 ± 1.82a	6.41 ± 1.45a	6.28 ± 2.4a	6.62 ± 1.78a
	Mouthfeel texture	7.26 ± 1.31a	7.41 ± 1.61a	7.40 ± 1.54a	7.30 ± 1.37a
	Flavor	6.78 ± 1.62a	6.82 ± 1.75a	6.76 ± 1.91a	7.04 ± 1.69a
	Overall acceptability	7.22 ± 1.53a	7.23 ± 1.38a	7.22 ± 1.56a	7.26 ± 1.54a
	The intensity of fermented fish smell	0.00 ± 0.00a	0.00 ± 0.00a	0.00 ± 0.00a	0.00 ± 0.00a

The data are presented as the mean ± standard deviation. Different letters in the same row indicate significant differences (*p* < 0.05).

## Data Availability

The original contributions presented in the study are included in the article, further inquiries can be directed to the corresponding author.
